# A Need for Consistency in Behavioral Phenotyping for ASD: Analysis of the Valproic Acid Model

**DOI:** 10.1155/2021/8863256

**Published:** 2021-03-19

**Authors:** Olivia Larner, Jane Roberts, Jeffery Twiss, Linnea Freeman

**Affiliations:** ^1^Neurosciences, Furman University, Greenville, SC, USA; ^2^Department of Psychology, University of South Carolina, Columbia, SC, USA; ^3^University of South Carolina Autism and Neurodevelopmental Disorders Excellence Initiative, Columbia, SC, USA; ^4^Department of Biological Sciences, University of South Carolina, Columbia, SC, USA; ^5^Department of Biology, Furman University, Greenville, SC, USA

## Abstract

Autism spectrum disorder (ASD) is a highly prevalent and impairing neurodevelopmental disorder that affects 1 : 54 persons. Over the last several decades, the reported incidence of ASD in the US has increased potentially due to increased awareness and improved diagnostic measurement. Although ASD prevalence is increasing, the etiology of ASD remains relatively unknown. To better understand the neurological basis of ASD, rodent models of ASD have been developed for research. Currently, there is not a standardized set of behavioral tests to quantify ASD-like behavior in rodents. The goal of this review is to present an overview of the methodologies used to analyze ASD-like behaviors in rodents, focusing on the valproic acid (VPA) model, and illustrate inconsistencies between different approaches. Despite that the *in utero* VPA rodent model for ASD is widely used and extensively characterized, behaviors vary substantially between different researchers. Moving forward, consistency in behavioral method analytics would benefit progress in evaluating interventions for all models of ASD and help to uncover unique qualities underlying mechanisms causing ASD signs and symptoms.

## 1. Introduction

Autism spectrum disorder (ASD) is a complex neurodevelopmental disorder defined by two main clusters of behaviors. The first group of behaviors, Cluster A, is defined by deficits in social communication and social interaction. The second group of behaviors, Cluster B, consists of repetitive patterns of behaviors, interests, and thoughts. ASD is described as a “spectrum” due to varying symptom presentation and severity among individuals with the disorder [1].

In 2016, the Autism and Developmental Disabilities Monitoring Network estimated ASD prevalence at 1 in 54 children aged 8 years, and ASD was 4.3 times more prevalent among boys compared to girls [2]. Over the last several decades, the reported incidence of ASD in the US has increased, potentially due to increased awareness and improved diagnostic assessments. Studies are also investigating the possibility that the increased prevalence of ASD is due to environmental factors influencing epigenetics [3]. Although ASD prevalence is increasing, the etiology of ASD remains relatively unknown.

To better understand the neurological basis of ASD, rodent models of ASD have been developed for research. Although “ASD” is often restricted to defining the human disorder, and animal models can only display autistic-like disorders, we cumulatively refer to the experimental animals with autistic-like features here as “ASD models”. In conjunction with neurological and cellular studies, rodents' behavior is evaluated through a variety of tests. Currently, there is not a standardized set of behavioral tests to quantify ASD-like behavior in rodents. Not only are different behavioral tests used but also the procedure and behavioral analysis for each test differs. Testing discrepancies include habituation periods, testing length, type of apparatus, quantification methods, behaviors analyzed, and more. Test variability makes it difficult to compare results across studies and may lead the researcher and reader to inappropriately implicate valproic acid (VPA) dose or delivery and species or strain for the differences.

The goal of this review is to give an overview of the methodologies used to analyze the behavioral tests measuring ASD-like behaviors in rodents, specifically the VPA model. These methodologies have contributed to a deeper understanding of ASD and improved characterization of ASD rodent models. We will focus on the Cluster A and Cluster B behaviors that characterize ASD as well as features of anxiety given its prevalence in ASD and that anxiety can be modeled via *in utero* administration of VPA.

## 2. Variation in the VPA Model

The VPA model of ASD is a widely used and extensively-studied model of ASD in rodents. VPA, also known as Depakote®, is commonly used to treat epilepsy and bipolar disorder in humans. In the 1990s, studies found that prenatal exposure to VPA through maternal ingestion resulted in the increased occurrence of ASD and other developmental disabilities in offspring [4]. Mechanistically, there is evidence to support that VPA's action as an HDAC inhibitor during neuronal development is responsible for the increased incidence of ASD [5]. Because prenatal VPA exposure is correlated with ASD in humans, *in utero* VPA exposure in rodents has been used to model ASD [6].

Within the VPA model, there is considerable variability in methods used to induce the model. One source of variation across studies is the gestational day at which VPA is administered. In Schneider and Przewłocki foundational research establishing the VPA model, pregnant rats were administered valproic acid on gestational day 12.5, which was specifically chosen to reflect human neuroanatomical changes in ASD [7]. Later, Kataoka et al. evaluated the effect of VPA administration in mice on gestational day 9, 12.5, and 14.5 and found that day 12.5 best correlated with behavioral and neuronal changes associated with ASD [5]. Although many studies follow the 12.5 gestational day guideline, others choose to give the VPA injection earlier on day 9 or 10, or later, on day 13 (Table 1). Timing of VPA administration is important because it will affect different stages of neuronal development. For example, the neural tube closes on embryonic day 11 in rats, so VPA exposure before closure could have a very different effect than after closure [7]. Research has also shown that late exposure, on day 14.5, does not decrease neuronal cell development in the prefrontal and somatosensory cortex to the same extent as exposure on day 12.5 [5]. It is important to highlight that the VPA model of autism in rodents is induced via one injection to the dam. In humans, VPA treatment would be administered orally likely before, during, and after the pregnancy. Furthermore, the majority of children with autism are not born to mother's taking VPA given the previous findings that VPA treatment increases autism incidence in their offspring as well as various teratogenic effects. The breadth of studies on VPA exposure to date has enhanced our understanding and characterization of the VPA rodent model of ASD. In a review by Mabunga et al., the proposed mechanisms and validity of the VPA rodent model of autism are described [6]. Briefly, some of the mechanisms for VPA action include histone deacetylase inhibition, increased glutamatergic neural density which leads to an excitatory/inhibitory imbalance, disruption of the maturation of serotonergic neurons, and increased reactive oxygen species [19–21]. Moving forward, it will be important to replicate and compare behavioral analyses between studies that utilize the same timing of embryonic exposure to ensure construct validity.

Another source of variation in the VPA model of ASD is the dose of VPA used. Currently, there is not a standardized VPA dose administered across studies. Schneider and Przewłocki first investigated prenatal exposure to 600 mg/kg of valproic acid [7]. Other studies have used as low as 200 mg/kg and as high as 800 mg/kg, but typically 500 mg/kg or 600 mg/kg is used (see Table 1). The reasoning for the 100 mg/kg difference is not clear. Both rodent and mice studies have used 500 mg/kg and 600 mg/kg. Additionally, routes of administration differ, as some studies use an intraperitoneal injection, while others use a subcutaneous injection (see Table 1). Future studies need to be conducted that control for these variables. Varying doses of VPA have contributed to our current understanding of this model as well as aspects of ASD. However, standardizing behavioral analyses across the different VPA doses will facilitate the comparison of results between different investigators and bring new understanding to behaviors that emerge in offspring due to VPA exposure.

The final variation in the VPA model is the use of male and female mice. In humans, there is approximately a four-fold increase in prevalence of ASD in males versus females [2]. Because of this sex effect, sex differences should be considered in rodent models of ASD, as sex-specific manifestations of ASD may occur [22]. Kataoka et al. demonstrated that prenatal VPA exposure differentially impacts neurodevelopment in both males and females [5]. The benefit of including both males and females in rodent ASD studies, using the VPA model as well as other models, has been extensively described in a review by Jeon et al. [19]. Despite this evidence, VPA studies continue to analyze male and female behavior together or exclusively use males (see Table 1). In order to better understand sex differences in ASD, future studies should investigate both males and females and data should be reported separately for each sex rather than aggregated.

## 3. Cluster A: Evaluation of Social Deficits

Cluster A behaviors in autism spectrum disorder are defined by deficits in social communication and social interaction. This includes deficits in social-emotional reciprocity, verbal and nonverbal communication, and difficulty making and maintaining relationships [1]. Several behavioral tests have been used to evaluate and quantify social communication and interaction in rodents. Although there are a few consistent behavioral tests, the definition of social behavior and procedures vary. The following provides an overview of different tests used to evaluate social behavior in the valproic acid model of ASD and highlights the different methodologies.

### 3.1. Three-Chamber Sociability Test

One commonly utilized assessment of social behavior is the three-chamber sociability test [23]. The apparatus for this test is divided into three chambers (left, middle, and right), with access open or restricted to each of the chambers. In the left and right chambers, a cylindrical cage or pencil cup-like container is placed to hold a “stranger” animal. The three-chamber sociability test comprises three phases. The first is a habituation phase to the apparatus. In the second phase, often referred to as the one-stranger test, a stranger mouse is placed in one of the cylindrical cages, and either the other cage serves as the “novel object” or a random object is placed in the cylinder. The subject rodent is then given the opportunity to explore all of the chambers for a set interval. During the last phase, known as the social novelty test, a new stranger is placed in the empty cylinder, and the previous stranger animal now becomes “the familiar.” Again, the test rodent is given the opportunity to explore all chambers for the same set interval.

Time and methods for the second and third phases of the three-chamber sociability test are generally consistent across studies (i.e., 10 minutes), but the habituation period differs most often from 5 to 10 minutes. Habituation of the test subject to the apparatus is either done to the center chamber only, all chambers, or both by the way of two habituation periods (Table 2). Habituation to all chambers can also occur with or without the cylindrical cages. Habituation to the center only allows both chambers to be completely novel during the testing period, but habituation to the entire chamber can rule out neophobia as a factor affecting sociability [16]. Habituation consistency is needed to substantiate comparisons across studies.

Studies using the VPA model have reported that the VPA rodents spend less time socializing than control rodents (see Table 2); however, how sociability is quantified also varies.

One way that sociability is measured is by the time spent in each chamber of the apparatus. Chamber time is typically recorded using a tracking software. Sometimes, chamber time is reported, but not considered in the actual measurement of sociability. Another way that sociability is quantified is through sniffing time. Sniffing time is typically scored manually. Some studies manually score sniffing in real-time, while others quantify sniffing time from video recordings (see Table 2). Another method used to evaluate sociability is by using an interaction zone approximately 3–5 cm radius around the cylinder or to measure contact time [24]. Interaction time is measured by analysis software or manually (see Table 2). Once sociability time is quantified, it must be compared across conditions, but how sociability is compared across studies differs. For the one-stranger test, sociability is either quantified by comparing time spent with the novel object to the time spent with the stranger rodent within the group or by comparing sociability time across each group. Sometimes, these comparisons are combined into a “social index.” The social index varies but typically includes time spent with the stranger rodent minus time spent with the novel object, divided by the total time spent with the stranger animal and novel object (see Table 2). The social novelty test analysis varies similarly (see Table 2). Overall, there are multiple ways to quantify and compare social behavior in the three-chamber sociability test.

Although human social behavior is undoubtedly different from rodent behavior, social quantification in rodents should attempt to use similar methods to those applied to humans to optimize translation. In humans, one way that social interaction is quantified is by coding the amount of time spent interacting with others and by oneself [25]. Thus, removing the attention from time spent with a novel object and simply focusing on time spent alone [26] would improve the alignment across fields and advance translation. Behavior analysis would include time spent with a “stranger mouse” compared to time spent alone as opposed to time spent with a “stranger mouse” compared to time spent with a novel object. Additionally, it is important for future rodent studies to distinguish between social interaction deficits that persist across interactions with familiar and unfamiliar rodents versus neophobia, which is usually typified by social withdrawal primarily during initial social encounters with novel partners as has been done in humans [27, 28]. The three-chamber sociability test is very useful and critical to characterizing changes to sociability in rodent models of autism. Furthermore, a number of data points can be obtained during this test, including socialization with a stranger rodent, socialization with a newly familiar rodent, time spent with a novel object, and time spent alone, as well as a number of additional behaviors: grooming, jumping, sniffing, etc. Given the number of outcome measures from this test, we argue that a minimum standard of analyses should be reported in order to better compare results between studies. For example, it is difficult to compare sociability outcomes between a study that reports a sociability index determined by time spent in each chamber versus a study that reports time spent sniffing the stranger rodent. Neither of these analyses is incorrect or invaluable; however, progress in assessing therapeutics could be limited by a lack of opportunity to compare outcomes across multiple studies. Instead, it would be beneficial to decide on specific outcome measures by a consortium of researchers, as has been done for many other fields. A standard for behavior analyses that take into account features of social deficits in humans with ASD, such as clearly reporting time spent with a stranger rodent and time spent alone, will improve the translation of findings, increase our understanding of how interventions affect social behavior, and contribute to the face validity of the model.

### 3.2. Social Interaction Test

Another test used to assess social behavior is the social interaction test, which typically consists of placing two unfamiliar rodents into an open field and evaluating their behavior [23]. Studies using the VPA model have reported a difference in social interaction in the VPA rodents, such as decreased sniffing time (see Table 3). Although studies report similar social deficits in the VPA model, there are noteworthy differences across studies. For example, the control rodent is not consistent. Some studies test one subject rodent and one unfamiliar rodent not included in the rest of the study [5, 10]. Other studies observe the interaction of one VPA rodent and one control (saline-injected) rodent [11], while others use two rodents in the same group [8]. These differences are important because a saline mouse may not interact with a VPA mouse the same way it interacts with a different saline mouse. For comparisons across studies, it is important to consider that the groups being tested might influence the results.

Another variation within the social interaction test is pretest isolation. Some studies house the test animals individually the night before testing [8, 11]. Alternatively, researchers have also chosen to individually house animals for four to five days before testing [5]. Individually housing the animals before testing could alter social motivation and influence the results [29]. In future studies, pretest isolation should be consistent.

The behaviors quantified in the social interaction test also vary. Studies have previously quantified a variety of behaviors such as sniffing, pinning, touching, and mounting (see Table 3). Furthermore, some behaviors were divided into locations such as sniffing of anogenital parts versus nonanogenital parts [8]. A uniform set of behaviors measured during the social interaction test would allow for more direct comparisons between studies.

### 3.3. Ultrasonic Vocalizations

In social situations, mice emit calls in the ultrasonic range as a form of communication. At present, there is limited research on rodent ultrasonic vocalizations (USVs). Research has shown that calls vary in frequency, length, and complexity, but the social motivations behind particular types of calls are still unknown. Although the specifics of rodent ultrasonic vocalizations are not yet fully understood, call analyses provide measurement of social communication. Since verbal communication deficits are prevalent in people with ASD, analyses of ultrasonic vocalizations may reinforce the model's validity [30].

There are several paradigms to facilitate rodent call production, since they do not emit ultrasonic vocalizations frequently in their home cage. The paradigms include pup separation from the mother, mating interactions with a male and a female in estrus, stranger-intruder, and urinary pheromone sniffing [30]. Pup isolation ultrasonic vocalizations in the VPA model have been recorded [10, 17, 31], but adult vocalizations in the VPA model have not yet been well-researched. Gandal et al. analyzed male ultrasonic vocalizations in a mating paradigm and found that the VPA males do not emit the same premating calls as the saline control [10]. Morales-Navas et al. evaluated both male and female infant Wistar rats. They reported significantly less calls in VPA-exposed offspring compared to controls, and male rats were affected significantly more than females. Control male rats revealed many more calls than any other group, suggesting baseline sex differences in USVs as well. VPA-exposed rats also revealed a decreased latency to their first call; however, no significant sex differences were determined for this measure [32]. Gzielo et al. have evaluated ultrasonic communication in male and female rats at three different time points: infant, adolescent, and adult. Both male and female rats exposed to VPA displayed a reduced number in calls as well as a shorter and elevated peak frequency. This research further determined that the adolescent time point may be more sensitive to sex differences due to the greatest effects in female rats exposed to VPA at this time point [33]. More research is emerging using USVs for social communication analysis, which will provide added detail to the complex alterations in socialization with ASD.

## 4. Cluster B: Evaluation of Repetitive Behavior

Cluster B behaviors in ASD are defined by restrictive, repetitive patterns of behavior, including motor stereotypes, fixed interests, and insistence on sameness [1]. Although repetitive behaviors are not the same in rodents as in humans, repetitive behaviors such as self-grooming, jumping, and digging have been studied that may parallel some behaviors in ASD individuals [30]. Although only a few repetitive behaviors are measured, the paradigm and environment, in which the behaviors are recorded, vary. The following provides an overview of different tests used to evaluate repetitive behavior in the VPA model of ASD and highlights the different methodologies.

### 4.1. Repetitive Behaviors in Open Environments

The paradigm and apparatus used to quantify repetitive behavior vary across studies (see Table 4). One method is to measure repetitive behavior in a standard cage in a novel environment. These studies typically last 10 minutes, but differ by whether the bedding is kept, removed, or replaced [10, 12, 14]. Other researchers have chosen to measure repetitive behavior over a longer period of time using the LABORAS vibration plate [14]. Another method is to measure repetitive behaviors in the open field, which allows simultaneous measurement of anxiety and repetitive behaviors. While variation in VPA dosage and timing has expanded our understanding of the ASD model, differences in conducting these behavioral tests combined with the varying models could stunt continued progress towards understanding the etiology of ASD as well as determining interventions.

### 4.2. Marble Burying

Another test used to measure repetitive behavior is the marble burying test. In the marble burying test, a set number of marbles are placed in a bedded cage. Mice are put in the cage for a set amount of time, and then the number of buried marbles is recorded [34]. Several studies have reported that the VPA rodents bury more marbles than the control (Table 5); however, the test procedures differ. One difference is total time, which varies from 10 minutes to 30 minutes. Another difference is the habituation period. Some studies habituated the rodent to the test cage before placing the marbles [12, 15], while others did not habituate the rodents at all [14].

### 4.3. Other Measures of Repetitive Behavior

Beyond marble burying and repetitive behavior in an open environment, a variety of tests have been used to quantify repetitive behavior. One test is the Y-maze, during which the rodent is allowed to move through the different arms of the maze. The arm alternations or repeated entries into a particular arm have been measured as restricted behavior [8, 16]. Another behavioral test used is the hole board test, in which the rodent is placed on an apparatus with evenly spaced holes across it. The number of times the animal dips its head into the holes is quantified as repetitive behavior [17]. In summary, many different methods are used to measure restrictive, repetitive behavior. A standardized version of the previously mentioned tests or a new, standardized battery would allow for cross-study comparisons to better quantify repetitive behaviors in rodent models of ASD.

## 5. Evaluation of Anxiety

Anxiety is one of the most common co-occurring disorders in people with ASD, especially in youths [35]. Because anxiety is highly prevalent in ASD, rodent models of ASD have also been closely evaluated for anxiety.

### 5.1. Open Field

Anxiety in the open field is a well-established paradigm and is quantified by analyzing the amount of time the rodent spends in the center of the field versus the periphery. The center of the field is anxiety-provoking because the animal is more exposed in the center, particularly to bright light, than by the protective walls [36]. In addition, the open field test is used to evaluate locomotor activity, which can be indicative of exploratory behavior [37]. Open field testing has been used by several studies to assess anxiety and locomotor activity in the VPA model of ASD (Table 6), but the findings vary across studies. Several studies have reported that the VPA rodents spend less time in the center of the field, indicative of anxiety [10, 11]. Conversely, others found no differences in center time between the VPA exposed and the control rodents [16]. Furthermore, findings vary in regard to locomotor activity. Some studies reported that the VPA rodents showed decreased overall motor activity [5, 10], while others have found increased motor activity [12]. Hirsch et al. utilized the open field to analyze both anxiety and repetitive behaviors. Spontaneous self-grooming was analyzed during a 10-minute test without habituation. Control and VPA-exposed rats revealed similar self-grooming during the first five minutes of the test; however, VPA-exposed rats displayed significantly greater self-grooming compared to control rats during the second half of the test [38].

The varying results are possibly due to the different methods used across studies. One critical difference was the duration of time in the open field, which varied from 10 to 90 minutes (see Table 6). Because animals' behavior changes over time due to habituation to the environment, comparing tests of different durations could precipitate misleading results. Additional factors that can lead to varying results across studies include sex, strain, and species (mouse versus rat) differences that are known to affect anxiety measures [39].

### 5.2. Elevated Plus Maze

The elevated plus maze is also a well-established test for anxiety behaviors. Anxiety is quantified by comparing the time the rodent spends in the closed arms versus the time spent in the open arms of the maze [40]. Several studies have used the elevated plus maze to measure anxiety in the VPA model (Table 7), but the results vary. Multiple studies have shown that the VPA rodents spend more time in the closed arms, indicative of anxiety [5, 8, 11]. Other studies have reported that the VPA rodents spent the same amount of time in the open and closed arms [16]. On the other hand, VPA mice have also been reported to spend more time in the open arms, which was concluded as the result of impulsive behavior [12]. Because the elevated plus maze is an established test, the procedures used across studies are the same. The varying results may be due to varying levels of anxiety, since anxiety does not always co-occur with ASD, impulsive behavior, or other outside factors.

## 6. Conclusion

The lack of consistency among various testing factors could lead to inconsistent outcomes across testing trials in the rodent models of ASD. On one hand, variable testing and analysis have added to the face validity and construct validity of ASD models, including the VPA model. However, variability in behavioral testing constrains comparisons across studies and may explain conflicting results between VPA and control animals. The body of work that has been conducted to date has contributed to a deeper understanding of both the VPA model as well as the behavioral tests used to define it. We chose to focus our review on the VPA model given our experience with this model and the breadth of studies for this model to date. However, we argue that the need for standardization in behavioral phenotyping applies to genetic models as well. Standardizing behavioral tests and analyses will allow for more direct comparisons between the various models aimed at modeling ASD, leading to a better understanding of the etiology of behavioral characteristics as well developing interventions.

We have identified the top three issues that we think need to be addressed for ASD behavioral phenotyping. First, potential sex differences need to be directly tested and consistently reported, including if there are no differences. In humans, ASD is more than four times more prevalent among males compared to females. For all ASD models, whether a sex bias is also prevalent in rodents needs to be fully explored. Studies need to be more transparent about their utilization of male vs. female rodents and preferably include both sexes for comparison. Second, repetitive behavior evaluation requires standardization. A consistent test for each repetitive behavior (i.e., grooming, digging, and sniffing) must be employed and reported. Currently, there is a great variability in which tests are conducted and how they are analyzed. We recommend a specific time for open field testing (such as 10 minutes), a standard open field apparatus (such as 76 × 76 cm), subjects tested individually, and subjects tested during a standardized time frame in order to avoid major variations in activity based on circadian rhythms. Third, sociability tests are more consistent between laboratories as compared to repetitive behavior tests, but these would also benefit from standardization of analyses between laboratories. A standard for isolation period, habituation period, and analytic methods would further improve comparisons between studies. For example, we recommend a 3-day isolation period before adult ultrasonic vocalization analysis and assessment of vocalizations between rodents of the same group (i.e., male VPA with male VPA rather than male VPA with male saline). Next, we recommend a minimum 10-minute habituation to the entire three-chamber sociability apparatus containing the cylindrical cages utilized to hold the stranger rodent (but without the stranger rodent during habituation) in order to decrease neophobia and focus on interaction with the stranger in the subsequent trials. Lastly, we recommend reporting time spent in each of the chambers as well as time interacting with “the stranger,” the empty cylindrical cage/novel object (during the second phase), and “the familiar” (during the third phase); variability in determining sociability index between research groups can make interpretation of results across multiple studies more difficult. While behavioral test variability in the past has contributed to a deeper understanding of the model itself, as well as features of behavioral tests, we argue that the next step will require clear comparisons between studies in order to develop interventions. The recommendations included here are possible standards, but we think the guidelines should be agreed upon by a consortium of researchers, as has been done for many other fields.

We recognize that current, as well as future ASD models, may not always determine statistically significant differences compared to controls on a standard battery. For example, an ASD model may reveal altered behaviors in Cluster A, but not Cluster B, or an ASD model may reveal altered behaviors in Cluster B, but no differences in anxiety measures. Our recommendation for behavior standardization should not limit these studies, but instead allow for better transparency about the utility and unique aspects of different ASD models. ASD is indeed a complex disorder with multiple characteristics, and it is not clear if the variation between the VPA models presented herein represent this spectrum or more technical differences of approach between different labs. If a model can highlight specific aspects of the disorder and then evaluate interventions for those characteristics, the field will have a clearer picture of the model as well as the intervention. Furthermore, this transparency will allow for improved comparisons between studies and between models.

For substantial progress to be made in studying rodent models of ASD, both implementations of a standardized behavioral battery with set methodologies need to be established as well as the introduction of carefully documented variation in the battery to provide meaningful, conclusive information that will shed critical light on potential interventions.

## Figures and Tables

**Table 1:** Valproic acid model and behavioral testing details.

Reference	Strain/species	VPA dose	Injection site	Time of exposure	Group numbers*∗*	Sex	Sexes analyzed separately?	Age behavioral testing	^#^ of different behavior tests
Schneider and Przewlocki [7]	Wistar rats	600 mg/kg	IP	E12.5	8 saline 8 VPA	Males only	—	P90–120	9
Markram et al. [8]	Wistar Han rats	500 mg/kg	IP	E12.5	136 saline 138 VPA	Males and females	No	P90	7
Dufour-Rainfray et al.[9]	Wistar rats	600 mg/kg	IP	E9	12 saline 12 VPA	Males only	—	P31	1
Gandal et al. [10]	C57BL/6Hsd mice	600 mg/kg	S.C.	E13	10–12 saline 10–12 VPA	Males and females	No	P35–75	7
Kataoka et al. [5]	ICR mice	500 mg/kg	IP	E12.5	18–24 saline 16–27 VPA	Males and females	Yes (female in supplementary)	P56	4
Chen et al. [11]	Sprague Dawley rats	500 mg/kg	IP	E12.5	21 saline 25 VPA	Males only	—	P28–30	3
Kim et al. [12]	ICR mice	300 mg/kg	S.C.	E10	10–12 saline 10–12 VPA	Not specified	—	P21–40	7
Bambini-Junior et al. [13]	Wistar rats	600 mg/kg	IP	E12.5	7 saline 12–19 VPA	Males only	—	P35–50	1
Kang and Kim [14]	C57BL6/J mice	600 mg/kg	S.C.	E13.5	11-12 saline 9-10 VPA	Not specified	—	P56–120	4
Baronio et al. [15]	Swiss mice	500 mg/kg	IP	E11	8 saline 8 VPA	Males only	—	P50	3
Campolongo et al. [16]	Outbred Cr1Fcen CF1 mice	600 mg/kg	S.C.	E12.5	21 saline 22 VPA	Males and females	Yes (female data unpublished)	P56	10
Bronzuoli et al. [17]	Wistar rats	500 mg/kg	IP	E12.5	5 saline 5 VPA	Males only	—	P35, 90	2
Sgritta et al. [18]	C57BL/6J mice	500 mg/kg	IP	E12.5	7–10 saline 7–10 VPA	Males only	—	P49–70	4

E, embryonic day; VPA, valproic acid; P, postnatal day; IP, intraperitoneal; S.C., subcutaneous; *∗*not including treatment groups.

**Table 2:** Three-chamber sociability test.

Reference	Habituation	Duration of each test	Coding methods	Sociability measurement	Index used?	Social behavior comparison	Outcome
Dufour-Rainfray et al. [9]	Not specified	3 min (only-2-stranger test)	Not specified	Time spent in each compartment and number of crosses from one compartment to another	No	Time spent in each chamber across groups	VPA spent more time in empty chamber. VPA had increased number of chamber crosses
Gantal et al. [10]	Full apparatus for 2 days before testing	10 min (only one stranger)	TopScan	Time spent sniffing and sniffing events	SPI = *S*1/(*S*1 + Obj)	Social preference index	VPA had reduced social preference
Kim et al. [12]	Center only for 5 min	10 min	EthoVision	Time spent in each chamber and approach sniffing time	SPI = *S*1/*E* SPI = *S*2 /*S*1	Time spent in each chamber compared across groups	VPA mice spent more time in the empty chamber and less time in stranger chamber. VPA spent more time with familiar mouse and less time with stranger
Bambini-Junior et al. [13]	Center only for 5 min	10 min	Manual real-time by two observers	Time spent in each chamber and time spent sniffing	SI = (*S*1 − Obj)/(*S*1 + Obj)SNI = (*S*2 − *S*1)/(*S*2 + *S*1)	Time with novel object and time with stranger rat	VPA spent equal time with novel object and stranger rat. VPA did not show novelty preference, but control preferred novel over familiar
Baronio et al. [15]	5 min (location not specified)	10 min	Manual real-time observation	Time spent in chamber and undefined time “spent exploring”	SI-(*S*1 − Obj)/(*S*1 + Obj)SNI = (*S*2 − F)/(*S*2 + *F*)	Time with stranger mouse and time with novel object	VPA spent equal time with novel object and stranger mouse. VPA spent equal time exploring novel and familiar mice, but control preferred novel over familiar
Kang and Kim [14]	Center only 10 min, full apparatus 10 min	10 min	EthoVision	Sniffing time and chamber time	PI = (*S*1 − *E*)/(*S*1 + *E*)PI = (*S*2 − *S*1)/(*S*1 + *S*2)	Time with stranger mouse and time with empty cage	VPA spent more time sniffing stranger than empty, but not as large of a difference as control. No social novelty preferences for VPA or control
Campolongo et al. [16]	5 minutes to full apparatus with cylinders	10 min (only one stranger)	Manual from recording	Sniffing time	No	Compared stranger mouse sniffing time across groups and to novel object	VPA spent less time sniffing stranger mouse than saline
Bronzuoli et al. [17]	Full apparatus 10 min	10 min (only one stranger)	Noldus Observer software	Sniffing time	No	Compared stranger sniffing time across groups	VPA spent less time sniffing stranger mouse than saline
Sgritta et al. [18]	Full apparatus 10 min	10 min	ANY maze, manual from recording (blind)	Sniffing time plus time climbing on cup	No	Time sniffing novel object and time sniffing stranger mouse	VPA spent same time sniffing stranger and object. VPA and control spent more time sniffing stranger mouse 2 than familiar

SI, sociability index; SNI, social novelty index; SPI, social preference index; S1, stranger/novel rodent 1; S2, stranger/novel rodent 2; F, familiar rodent; Obj, novel object; E, empty chamber.

**Table 3:** Social interaction test.

Reference	Pretest habituation	Length	Coding methods	Nonsubject animals	Apparatus notes	Behaviors analyzed	Outcome
Markram et al. [8]	Individually housed the night before	20 min	Not specified	Same group, sex, and weight	Contained an “escape” tube large enough for one rat	Pinning, touching, grooming each other, sniffing of anogenital parts and nonanogenital parts	VPA decreased pinning nonanogenital sniffing, and touching bouts. VPA increased hiding time
Kataoka et al. [5]	Resident habituated to cage for 60 min	20 min	Manual from recording (blind)	Unfamiliar ICR mouse, same age	Typical cage	Sniffing, allogrooming, aggression	VPA males decreased sniffing time. VPA females increased sniffing time
Chen et al. [11]	Individually housed the night before	20 min	EthoVision	One VPA-exposed and one saline-exposed; same age, sex, and weight	White plastic box	Sniffing, mounting, grooming	VPA males decreased sniffing frequency and duration
Sgritta et al. [18]	—	Not stated	Manual using ANY-maze (blind)	Age, sex, and treatment matched	Plexiglass box	Following, touching, sniffing, grooming, crawling	Results not reported for VPA

**Table 4:** Repetitive behavior in open environments.

Reference	Apparatus	Pretest habituation	Duration	Coding methods	Behaviors analyzed	Outcome
Gandal et al. [10]	Cage without bedding	20 min to new cage	10 min	Manual real-time (blind)	Time spent grooming	VPA increased total grooming time
Kim et al. [12]	Typical cage	10 min to new cage	10 min	Real-time 2 meters from cage	Grooming and digging	VPA increased digging. No difference for grooming
Kang and Kim [14]	Fresh bedded home cage in novel environment	72-hour isolation	10 min	Manual from recording	Grooming time, jumping bouts, digging	VPA increased grooming time and jumping bouts
Kang and Kim [14]	48-hour movements in familiar environment	48-hour isolation	48 hours	Vibration sensitive plate LABORAS cage	Distance moved, grooming time, total rearing time	No difference between VPA and saline
Campolongo et al. [16]	Clear plexiglass cylinder	1-hour habituation for 2 days before testing	10 min	Manual from recording, condition blind	Grooming of all body regions	Effect of VPA treatment on grooming

**Table 5:** Marble burying test.

Reference	Pretest habituation	Bedding (cm)	Marbles	Duration (minutes)	Outcome
Kim et al. [12]	10 min to testing cage without marbles	3	20 glass	20	VPA buried more marbles
Kang and Kim [14]	Habituation to room only	4	21 stainless steel or glass	30	No difference between VPA and control
Baronio et al. [15]	10 min to testing cage without marbles	4	20 black	10	VPA buried more marbles

**Table 6:** Open field.

Reference	Pretest habituation	Duration (minutes)	Coding methods	Behaviors analyzed	Center area	Outcome
Gandal et al. [10]	—	10	TopScan	Total distance traveled and time spent in the center of field	19% of total area	VPA spent less time in the center
Kataoka et al. [5]	—	90	Acti-track	Distance traveled, number of rearings, and number of entries into the center	Center undefined	VPA male and female decreased distance traveled, rearings, and entries into the center
Chen et al. [11]	—	15	EthoVision	Present time in central zone and total distance moved	25% of total area	VPA spent less time in the center
Kim et al. [12]	5 min	20	EthoVision	Total distance and velocity	—	VPA had increased motor activity and velocity
Campolongo et al. [16]	—	30	ANY-maze and manual (blind)	Total distance traveled, time spent in the center, time grooming, and number of rearings	26% of total area	No difference in the center time for VPA and saline. Treatment effect on grooming time
Sgritta et al. [18]	—	10	Any-maze	Distance traveled, speed, and time spent in the center	25% of total area	Results not reported for VPA

**Table 7:** Elevated plus maze.

Reference	Behaviors analyzed	Coding methods	Duration (min)	Outcome
Markram et al. [8]	Time spent in open and closed arms, total distance, and total velocity	Not specified	5	VPA spent less time in open arms than control. Distance and velocity were the same
Kataoka et al. [5]	Time spent and entries into open and closed arms	Manually scored from video (blind)	5	VPA males and females spent less time in open arms and more time in closed arms compared to control
Chen et al. [11]	Percent time spent in each arm and total distance	EthoVision	5	VPA males spent less time in open arms compared to control
Kim et al. [12]	Relative stay time ratio (open/closed)	EthoVision	5	VPA spent more time in open arms
Campolongo et al. [16]	Time spent in arms, distance traveled in arms, rearing, grooming, and head dips	ANY-maze for locomotion, manual for behavioral (blind)	5	VPA and saline spent same amount of time in open and closed arms

## Data Availability

No data were used to support this study.
